# Novel and Functional DNA Sequence Variants within the *GATA6* Gene Promoter in Ventricular Septal Defects

**DOI:** 10.3390/ijms150712677

**Published:** 2014-07-17

**Authors:** Chunyu Li, Xianke Li, Shuchao Pang, Wei Chen, Xianyun Qin, Wenhui Huang, Changqing Zeng, Bo Yan

**Affiliations:** 1 Division of Electrocardiogram, Jining Medical University Affiliated Hospital, Jining Medical University, Jining 272029, China; E-Mail: llichunyu@163.com; 2 Division of Anesthesia, Jining First People’s Hospital, Jining 272011, China; E-Mail: 13963722217@163.com; 3 Shandong Provincial Key Laboratory of Cardiac Disease Diagnosis and Treatment, Jining Medical University Affiliated Hospital, Jining Medical University, Jining 272029, China; E-Mails: tonyp0720@126.com (S.P.); qinxianyun@126.com (X.Q.); huangwenhui1986@163.com (W.H.); 4 Laboratory of Genome Variation and Precision Biomedicine, Beijing Institute of Genomics, Chinese Academy of Sciences, Beijing 100101, China; E-Mail: chenw@big.ac.cn

**Keywords:** congenital heart disease, ventricular septal defect, *GATA6*, promoter, DNA sequence variants

## Abstract

Congenital heart disease (CHD) is the most common birth defect in humans. Genetic causes and underlying molecular mechanisms for isolated CHD remain largely unknown. Studies have demonstrated that GATA transcription factor 6 (*GATA6*) plays an essential role in the heart development. Mutations in *GATA6* gene have been associated with diverse types of CHD. As *GATA6* functions in a dosage-dependent manner, we speculated that changed *GATA6* levels, resulting from DNA sequence variants (DSVs) within the gene regulatory regions, may mediate the CHD development. In the present study, *GATA6* gene promoter was genetically and functionally analyzed in large groups of patients with ventricular septal defect (VSD) (*n* = 359) and ethnic-matched healthy controls (*n* = 365). In total, 11 DSVs, including four SNPs, were identified in VSD patients and controls. Two novel and heterozygous DSVs, g.22169190A>T and g.22169311C>G, were identified in two VSD patients, but in none of controls. In cultured cardiomyocytes, the activities of the *GATA6* gene promoter were significantly reduced by the DSVs g.22169190A>T and g.22169311C>G. Therefore, our findings suggested that the DSVs within the *GATA6* gene promoter identified in VSD patients may change *GATA6* levels, contributing to the VSD development as a risk factor.

## 1. Introduction

Congenital heart disease (CHD) is the most common human birth defects affecting 1%–2% of live birth [1]. In the past two decades, genetic studies and animal experiments have revealed a number of CHD-associated genes, including GATA factor 4 (*GATA4*), T-box transcription factor 5 (*TBX5*) and NK2 transcription factor related-locus 5 (*NKX2-5*) [2,3]. However, genetic causes and underlying molecular mechanisms for isolated CHD, which account for a majority of CHD cases, remain largely unknown. *GATA6* is a member of GATA transcription factor family containing a highly conserved DNA-binding domain. During the embryonic development, GATA factors regulate the cell differentiation, proliferation and survival. *GATA1*, *GATA2* and *GATA3* genes are expressed in hematopoietic stem cells and related derivatives. *GATA4*, *GATA5* and *GATA6* genes are expressed in various tissues derived from mesoderm and endoderm. In the developing heart, *GATA4*, *GATA5* and *GATA6* genes are expressed in a partial overlapping pattern [4,5,6].

*GATA6* gene is expressed in developing heart and continues to be expressed in the adult cardiomyocytes in human and experimental animals [7,8]. In mouse embryos, *GATA6* gene is expressed in the precardiac mesoderm, heart tube, atria and ventricles [7,9]. *GATA6* plays an important role in endocardial cushion formation and outflow tract morphogenesis [10]. Targeted disruption of *GATA6* gene in mice leads to embryonic lethality with defective endodermal differentiation [11]. Mice with heterozygous deletion of *GATA6* gene develop normally [12]. Cardiomyocyte-specific deletion and over-expression experiments have indicated that *GATA6* gene is required for cardiac hypertrophic response and differentiated gene expression in myocytes [13]. Tissue-specific inactivation of *GATA6* gene in vascular smooth muscles or neural crest causes cardiovascular defects, including interrupted aortic arch and persistent truncus arteriosus [14]. In addition, *GATA6*, like *GATA4*, can direct the efficient generation of cardiomyocytes from embryonic stem cells [15]. Therefore, *GATA6* is a critical regulator in the heart development.

*GATA6* gene mutations have been reported in familial and isolated CHD patients in different ethnic populations, including atrial septal defect, atrioventricular septal defect, persistent truncus arteriosus, tetralogy of Fallot and ventricular septal defect (VSD) [16,17,18,19,20,21,22,23,24,25]. Mutations in *GATA6* gene include missense mutations, deletions and copy number variants. *GATA6* gene mutations have also been found in patients with diabetes and pancreatic agenesis [26,27,28]. To date, *GATA6* gene mutations found in CHD patients are located in the coding regions and splicing sites, regulatory regions of *GATA6* gene have not been studied and reported. *GATA6* has been demonstrated to act in a dosage-dependent manner in the heart [12,29]. Thus, we speculated that the DNA sequence variants (DSVs) within the *GATA6* gene regulatory regions may alter *GATA6* levels and mediate the CHD development. In the present study, promoter region of the human *GATA6* gene was genetically and functionally analyzed in large groups of VSD patients and healthy controls.

## 2. Results and Discussion

### 2.1. DNA Sequence Variants (DSVs) Identified in Ventricular Septal Defect (VSD) Patients and Controls

**Figure 1:**
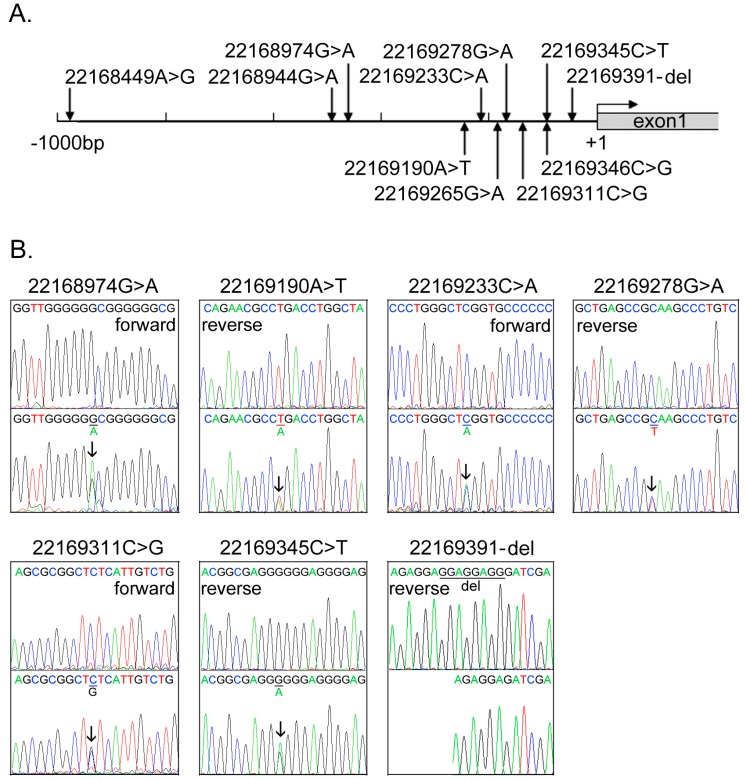
The DSVs within the *GATA6* gene promoter identified in VSD patients and controls. (**A**) Schematic representation of the identified *GATA6* gene DSVs. The DSVs were named according to their locations in the *GATA6* genomic sequences (NCBI: NC_000018.10). The transcription starts at 22169443 in the first exon that is untranslated; (**B**) Chromatograms of the seven novel and heterozygous DSVs. Sequencing orientations are indicated as forward or reverse. Top panels show wild type and bottom panels heterozygous DSVs. DSVs are marked with arrows and deletion is underlined. The heterozygous deletion DSV, g.22169391-del, was confirmed by subcloning the DNA fragments into expression vector pGL3-basic and directly sequenced.

The *GATA6* gene promoters were bi-directionally sequenced in VSD patients (*n* = 359) and healthy controls (*n* = 365). In total, 11 DSVs, including four single-nucleotide polymorphisms (SNPs) were identified, distributions of which were summarized in Table 1. The locations of the DSVs were indicated in Figure 1A. Chromatograms of the novel DSVs were shown in Figure 1B. Two novel heterozygous DSVs, g.22169190A>T and g.22169311C>G, were identified in two VSD patients, but in none of controls. The DSV g.22169190A>T was found in a two-year-old boy with a membranous VSD and the DSV g.22169311C>G in a 5-year-old boy with a muscular VSD. Four novel and heterozygous DSVs, g.22168974G>A, g.22169233C>A, g.22169278G>A and g.22169391-del, were only found in three controls. The deletion DSV, g.22169391-del, was confirmed by subcloning the DNA fragment into an expression vector and direct sequencing. One novel and heterozygous DSV, g.22169345C>T, and four SNPs, g.22168449A>G (rs189133474), g.22168944G>A (rs144923558), g.22169265G>A (rs146748749) and g.22169346C>G (rs139399350), were identified in both VSD patients and controls with similar frequencies. In addition, the SNPs, g.22168944G>A (rs144923558) and g.22169265G>A (rs146748749), were in complete linkage disequilibrium (*D*’ = 1, *r*^2^ = 1) in this study population.

**Table 1:** GATA transcription factor 6 (*GATA6*) gene promoter DNA sequence variants (DSVs) in ventricular septal defect (VSD) patients and controls.

DSVs	Genotype	Location ^a^	Controls ( *n* = 365)	VSD ( *n* = 359)	*p* Value
g.22168449A>G (rs189133474)	AG	−994 bp	10	6	0.329
g.22168944G>A (rs144923558)	GG	−499 bp	347	333	0.212
	GA		18	24	
	AA		0	2	
g.22168974G>A	GA	−469 bp	1	0	–
g.22169190A>T	AT	−253 bp	0	1	–
g.22169233C>A	CA	−210 bp	1	0	–
g.22169265G>A (rs146748749)	GG	−178 bp	347	333	0.212
	GA		18	24	
	AA		0	2	
g.22169278G>A	GA	−165 bp	1	0	–
g.22169311C>G	CG	−132 bp	0	1	–
g.22169345C>T	CT	−96 bp	5	7	0.541
g.22169346C>G (rs139399350)	CC	−97 bp	316	313	0.330
	CG		48	43	
	GG		1	3	
g.22169391-del	CCTCCTCC/-	−45–52 bp	1	0	–

^a^, DSVs were located upstream to the transcription start site (22169443, NC_000018.10); –, not compared.

### 2.2. Functional Analysis of the DSVs

**Figure 2:**
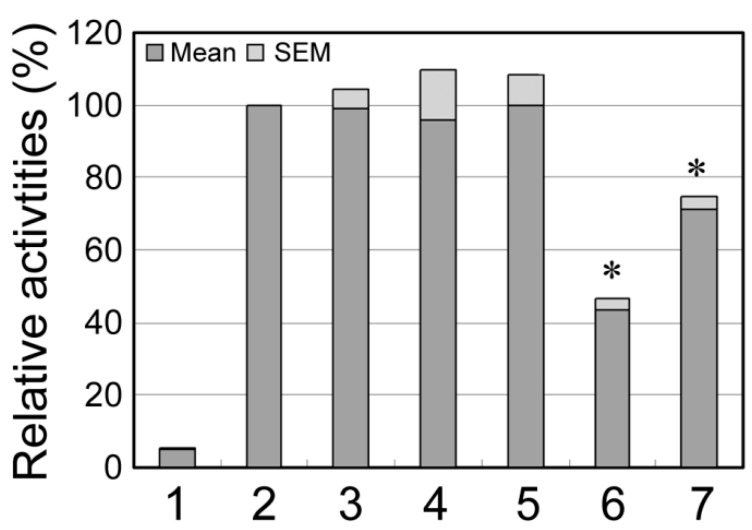
Relative transcriptional activities of the wild type and variant *GATA6* gene promoters. *GATA6* gene expression constructs were transfected into H9c2 cells and dual-luciferase activities were measured. The transcriptional activity of wild type *GATA6* gene promoter was designated as 100%. The data were represented as mean ± SEM from three independent transfection experiments, in triplicate. Lanes 1, pGL3-basic, a negative control; 2, WT, wild type; 3, pGL3-22168449G; 4, pGL3-22168944A; 5, pGL3-22168974A; 6, pGL3-22169190A>T; 7, pGL3-22169311G.*, *p* < 0.001, compared to pGL3-WT.

The two novel DSVs identified in VSD patients, g.22169190A>T and g.22169311C>G, were analyzed with TFSEARCH program (http://www.cbrc.jp/research/db/TFSEARCH.html) [30]. The results suggested that the DSV g.22169190A>T may abolish a putative binding site for retinoid-related orphan receptor alpha 1. The DSV g.22169311C>G did not affect binding site for known putative transcription factors. To examine their transcriptional activities, expression constructs containing wild type (pGL3-WT) and variant *GATA6* gene promoters (pGL3-22168449G, pGL3-22168944A, pGL3-22168974A, pGL3-22169190T and pGL3-22169311G.) were generated. The constructs were transfected into H9c2 cells and dual-luciferase activities were measured. The results showed that the DSVs, g.22169190A>T and g.22169311C>G, significantly reduced the transcriptional activities of the *GATA6* gene promoter (Figure 2). In contrast, the DSV, g.22168974G>A, and two SNPs, g.22168449A>G (rs189133474) and g.22168944G>A (rs144923558), did not significantly change the *GATA6* gene promoter activity.

### 2.3. Discussion

Misregulation of gene expression programs has been implicated in a broad range of human diseases, including cancer, inflammation, diabetes and cardiovascular diseases [31]. A set of de novo mutations in histone modification-related genes, which affect levels of transcriptional outputs, has been found in CHD patients [32]. Two loci have been identified in patients with secundum atrial septal defect with genome-wide association studies [33]. In the present study, we identified two novel heterozygous DSVs within the *GATA6* gene promoter in two VSD patients, which were found in none of controls. In cultured cardiomyocytes, these DSVs significantly reduced the transcriptional activities of the *GATA6* gene promoter. Therefore, these *GATA6* gene DSVs may mediate the DSV development as a risk factor.

Human *GATA6* gene has been localized to chromosome 18q11.1-q11.2 [8]. In human, *GATA6* is mainly expressed in fetal heart and lung. *GATA6* is also expressed in adult heart, pancreas, ovary, lung, liver, central nervous system, adrenal and vascular smooth muscle cells [8,34,35]. Although the human *GATA6* gene promoter has not been characterized, the 5' upstream region (~1.2 kb) of the mouse *GATA6* gene has been shown to be necessary for its heart-specific expression [9,36]. A mouse *GATA6* cardiac enhancer has been identified, which is directly activated by *NKX2-5* [37]. Reduced or elevated *GATA6* gene expression has been reported in patients with neonatal lung diseases, pulmonary arterial hypertension, pancreatic carcinoma, malignant astrocytoma and polycystic ovary syndrome [38,39,40,41,42]. The two *GATA6* gene DSVs identified in this study, located at −132 and −253 bp upstream to the transcription start site, may change *GATA6* levels during the heart development.

In the heart development, many networks of cardiac transcription factors, cofactors and chromatin regulators are strictly interacted and regulated [43,44,45,46]. Animal studies have demonstrated that *GATA6* regulates and interacts with a number of critical transcription factors and structural molecules. *GATA6* interacts with *GATA4* to synergistically regulate atrial natriuretic factor (*ANF*) and B-type natriuretic peptide (*BNP*) gene expression by binding to their promoters [47]. Loss of both Gata4 and Gata6 leads to acardia in mice [48]. *GATA6* interacts with *GATA5* in endocardial cushion formation and outflow tract development. Compound loss of a *GATA5* and a *GATA6* allele leads to double outlet right ventricle and VSD [10]. *GATA6* and *TBX5* synergistically activate *ANF* gene in the developing heart [49]. *NKX2-5* gene expression is regulated by a number of cardiac-specific enhancers containing GATA binding sites [50,51]. Heterozygous loss of *GATA6* gene result in reduced expression of *NKX2-5* gene [10]. In addition, Hey basic helix-loop-helix transcription factor 2 (Hey2), a downstream effector of Notch signaling, binds to *GATA6* and directly represses *ANF* gene expression [52]. Mice lacking Hey2 develop VSD and cardiomyopathy [53]. Therefore, altered levels of *GATA6* may contribute to the CHD development by interfere with cardiac gene regulatory networks.

## 3. Materials and Methods

### 3.1. Patients and Controls

All VSD patients (*n* = 359, male 184, female 175, age range from 3 months to 42 years, median age 4.17 years) were recruited from Division of Cardiac Surgery, Jining Medical University Affiliated Hospital, Jining Medical University, Jining, Shandong, China. The VSD patients were diagnosed with three-dimensional echocardiography and further confirmed with cardiac surgery or cardiac catheterization. In this cohort of VSD patients, none had familial CHD history. Ethnic-matched healthy controls (*n* = 365, male 304, female 61, age range from one month to 39 years, median age 3.67 years) were from the same hospital. This study (NSFC 81370271, 2013) was approved by the Human Research Ethics Committee of Jining Medical University Affiliated Hospital. Informed consents were obtained from patients and guardians.

### 3.2. Sequence Analysis

Leukocytes were isolated from vein blood. Genomic DNAs were extracted with QIAamp DNA mini kit (QIAGEN, Valencia, CA, USA). *GATA6* PCR primers were designed based on genomic sequence of the human *GATA6* gene (NCBI: NC_000018.10). *GATA6* gene promoter of 1315 bp (from −1246 to +69 bp to the transcription start site) was sequenced. Two overlapping DNA fragments, 695 bp (−1246~−551 bp) and 663 bp (−594p~+69 bp), were generated with PCR. The sequences and locations of the PCR primers were shown in Table 2. The PCR products were bi-directionally sequenced with 3500 Genetic Analyzer (Applied Biosystems, Foster City, CA, USA). DNA sequences were aligned and compared with the wild type sequence of the *GATA6* gene promoter.

**Table 2:** PCR primers for the human *GATA6* gene promoter ^a^.

Primers	Sequences	Location	Products
Sequencing			
*GATA6*-F1	5'-ACCAGAGCCTAAACGCTTTC-3'	22168197	695 bp
*GATA6*-R1	5'-ACCCTATCTCGGGATGCTAC-3'	22168891	
*GATA6*-F2	5'-CCGAAACCACCACGACCTGAG-3'	22168849	663 bp
*GATA6*-R2	5'-TGGGCTCCTGATTGGACTCACC-3'	22169511	
Functioning			
*GATA6*-F	5'-(KpnI)-ACGCCTCTTGTCCTAAAGTCTC-3'	22168318	1173 bp
*GATA6*-R	5'-(HindIII)-CGAGCCCTAAACAAACAGC-3'	22169490	

^a^, PCR primers were designed based on the genomic DNA sequence of the human *GATA6* gene. (NC_000018.10). The transcription start site (+1) is at position of 22169443.

### 3.3. Functional Analysis

The DNA fragments of wild type and variant *GATA6* gene promoters (1173 bp, from −1125 to +48 bp) were generated by PCR. As shown in Table 1, a KpnI site was added to the *GATA6* forward primer and a HindIII site to the *GATA6* reverse primer. Expression constructs were generated by subcloning PCR products into KpnI and HindIII sites of a reporter vector-pGL3-basic, expressing luciferase gene. To examine the activities of the *GATA6* gene promoters, designated expression constructs (2.0 μg) were transiently transfected into rat cardiomyocyte cells (H9c2) in 6-well plates. The transfected cells were collected 48 h posttransfection. Expression construct expressing renilla luciferase gene (*pRL-TK*) (40 ng) was used as an internal control. Empty vector pGL3-basic was used as a negative control. Luciferases activities were measured using dual-luciferase reporter assay system on a Glomax 20/20 luminometer (Promega, Madison, WI, USA). The transcriptional activities of the gene promoter were represented as ratios of luciferase over renilla luciferase activities. All the experiments were repeated at least three times independently, in triplicate.

### 3.4. Statistical Analysis

Quantitative data were represented as mean ± SEM and compared by a standard Student’s *t*-test. Frequencies of the DSVs within the *GATA6* gene promoter in VSD patients and controls were compared with SPSS v13.0 (SPSS, Chicago, IL, USA). A *p* < 0.05 was considered as statistically significant.

## 4. Conclusions

In conclusion, two novel and heterozygous DSVs within the *GATA6* gene promoter were identified in VSD patients, which have transcriptional activities in cultured cardiomycytes. The findings suggested that these *GATA6* DSVs may be involved in VSD formation by changing *GATA6* levels as a risk factor. As morbidity and mortality in adult CHD patients are significantly higher than general populations, likely due to genetic defects [54,55], genetic studies may provide insight into designing novel therapies for adult CHD patients.
